# Interpreting pathology test result values with comparators (<, >) in Electronic Health Records research: an OpenSAFELY short data report

**DOI:** 10.12688/wellcomeopenres.19858.1

**Published:** 2023-11-22

**Authors:** Helen J Curtis, Louis Fisher, David Evans, OpenSAFELY Collaborative, Sebastian Bacon, Amir Mehrkar, Ben Goldacre, Brian MacKenna

**Affiliations:** 1 Nuffield Department of Primary Care Health Sciences, University of Oxford, Oxford, England, OX2 6GG, UK

**Keywords:** Pathology, electronic health records, kidney function tests

## Abstract

**Background:**

Numeric results of pathology tests are sometimes returned as a range rather than a precise value, e.g. “<10”. In health data research, test result values above or below clinical threshold values are often used to categorise patients into groups; however comparators (<, > etc) are typically stored separately to the numeric values and often ignored, but may influence interpretation.

**Methods:**

With the approval of NHS England we used routine clinical data from 24 million patients in OpenSAFELY to identify pathology tests with comparators commonly attached to result values. For each test we report: the proportion returned with comparators present, split by comparator type and geographic region; the specific numeric result values returned with comparators, and the associated reference limits.

**Results:**

We identified 11 common test codes where at least one in four results had comparators. Three codes related to glomerular filtration rate (GFR) tests/calculations, with 31-45% of results returned with “≥” comparators. At least 90% of tests with numeric values 60 and 90 represented ranges (≥60 and ≥90 respectively) rather than exact values. The other tests - four blood tests (Nucleated red blood cell count, Plasma C reactive protein, Tissue transglutaminase immunoglobulin A, and Rheumatoid factor), two urine tests (albumin/microalbumin) and two faecal tests (calprotectin and quantitative faecal immunochemical test) - were returned with “≤” comparators (29-86%).

**Conclusions:**

Comparators appear commonly in certain pathology tests in electronic health records. For most common affected tests, we expect there to be minimal implications for researchers for most use-cases. However, care should be taken around whether results falling exactly on clinical threshold values should be considered “normal” or “abnormal”. Results from GFR tests/calculations cannot reliably distinguish between mild kidney disease (60-<90) versus healthy kidney function (90+). More broadly, health data researchers using numeric test result values should consider the impact of comparators.

## Introduction

OpenSAFELY is a new electronic health records platform used for research into COVID-19 for patients registered with general practices in England. Pathology tests results recorded in general practice, such as blood or urine tests, can give us important information about patients’ health, including various aspects of research related to COVID-19. These include: grading the severity of patients’ pre-existing conditions (which may impact their risk from COVID-19) ^1^; monitoring changes in health condition (for example after contracting COVID-19); or assessing healthcare service provision (e.g. whether people with diabetes generally experienced worsening disease during the COVID-19 pandemic).

Researchers may apply clinical threshold values to classify patients’ numeric result values into severity categories, for example chronic kidney disease ^2^. However, for some tests, result values can be reported as a range, indicated by a "less than" or "greater than" comparator, e.g. "<10". This may be due to the measurement limits of the machines used to analyse the sample, or to simply report a “normal” result by referring to a clinical threshold. For some tests this type of result is very common, but comparators are stored separately to the numeric result values in electronic health records, and often not taken into consideration when researchers extract test results. This means that values could potentially be grouped incorrectly, e.g. "<10" would be taken as "10" and incorrectly grouped with values "≥10". Similarly, it may be impossible to determine how a given value relates to a clinical threshold e.g. if grouping patients as above or below a clinical threshold of 5, a value reported as "<10" could fall in either group.

We therefore set out to find the most affected tests, how often they have comparators and which direction. We also identify the numeric values most often associated with comparators and compare them with reference limits, in order to inform suitable threshold values for researchers. This report is intended to support all researchers and studies carried out in OpenSAFELY-TPP informing responses to the COVID-19 pandemic, and those working with similar data elsewhere.

## Methods

### Study design

Retrospective cohort study across 24 million patients registered with English general practices in OpenSAFELY-TPP.

### Data Source

All data were stored and analysed securely using the OpenSAFELY platform, https://www.opensafely.org/, as part of the NHS England OpenSAFELY COVID-19 service. The dataset analysed within OpenSAFELY-TPP is based on GP surgeries using TPP SystmOne software. Data include pseudonymised data such as coded diagnoses, medications and physiological parameters. No free text data are included. All code is shared openly for review and re-use under MIT open license ( https://github.com/opensafely/pathology-comparators-short-report). Detailed pseudonymised patient data is potentially re-identifiable and therefore not shared. Detailed pseudonymised patient data is potentially re-identifiable and therefore not shared.

### Codelists

We conducted an initial analysis over a sample period of three days to identify a subset of common tests (≥50 records per day), and where ≥25% of the records had comparators. Eleven test codes met these criteria ( Table 1); three of which were for Glomerular filtration rate (GFR) tests, four codes for other blood tests, two urine and two faecal tests.

**Table 1.** Tests included by code (SNOMED code) and term (SNOMED description). Codes are grouped into four types and the common use of the test is shown.

code	term	type	Common use
1011481000000105	EGFR calculated using creatinine CKDEC equation	GFR	Reduced levels indicate kidney disease
1020291000000106	GFR calculated by abbreviated MDRD		
80274001	Glomerular filtration rate		
1022461000000100	Nucleated red blood cell count	Other blood tests	Raised levels indicate hypoxia / possible malignancy
999651000000107	Plasma C reactive protein		Raised levels indicate inflammation / possible infection
1013671000000106	Tissue transglutaminase immunoglobulin A level		Raised levels indicate coeliac disease / other gluten sensitivities
992081000000101	Rheumatoid factor		Raised levels indicate autoimmune diseases such as rheumatoid arthritis
1003301000000109	Urine albumin level	Urine	Raised levels indicate kidney disease or risk thereof
1010251000000109	Urine microalbumin level		
1049361000000101	Quantitative faecal immunochemical test	Faecal	Raised levels indicate bowel disease
1002571000000102	Faecal calprotectin content		Raised levels indicate bowel inflammation

*(E)GFR = (Estimated) Glomerular filtration rate* *CKDEC = Chronic Kidney Disease Epidemiology Collaboration equation per 1.73 square metres* *MDRD = Modification of Diet in Renal Disease Study Group calculation*

### Study population

We included patients of all ages registered with a GP in OpenSAFELY-TPP as of the start of each week over a two-week period beginning 1st October 2021, and having any test in the codelist.

### Study measures

For each code in the codelist we counted the number of patients with a code recorded per week with an associated non-zero numeric result value. Zeros usually represent non-numeric results; for some tests zero values are possible but these cannot be distinguished. Limiting to non-zero values ensures we count those returned with a valid value only. For the latest result per patient per week, we extracted the numeric result value (available to one decimal place), and the comparator associated with the result. We also extracted the upper and lower reference bounds returned alongside the result; these represent the upper and lower limit for what is considered a “normal” result; however some tests only have one limit (e.g. a test for which any low value is “normal” and an “abnormal” result is only defined as being above a clinical threshold). Tests may have multiple possible upper and/or lower limits as they can vary by laboratory and patient factors such as age. Counts were rounded to the nearest 10. Percentages were calculated after rounding.

***Overall rate of comparators present per test.*** We grouped tests with no comparator, “=” or “~” together as the no-comparator group. We grouped tests with “>” or “≥” together as “≥”, and similarly for “<” and “≤”. We report the proportion of each test which were returned with comparators present.

***Values associated with comparators.*** We identified the numeric values most commonly associated with comparators in order to ascertain the important cut-off points for users studying these results. For each test, we identified numeric values with a total count >100 and ≥0.1% returned with comparators present, limited to the main comparator in use for each test (≥ or ≤). We report the proportion of each test result value which were returned with comparators present and compare these values to the most common upper or lower reference bounds (as applicable) for the test.

***Regional variation.*** We report the proportion of each test which were returned with comparators present by the NHS region of the practice at which each patient was registered. Some regions do not have full population coverage in the cohort as only practices using TPP software are included.

### Software and reproducibility

Data management and further analysis were performed using Python 3. Code for data management and analysis, as well as codelists, are archived online https://github.com/opensafely/pathology-comparators-short-report.

### Patient and public involvement

OpenSAFELY has developed a publicly available website https://www.opensafely.org/ through which they invite any patient or member of the public to make contact regarding the broader OpenSAFELY project.

## Results

### Overall rate of comparators present per test

We included 461,430 tests over the two-week study period, 166,800 (36.1%) of which had results that were associated with a comparator ( Table 2). GFR tests/calculations typically had ≥ comparators (31–45% of tests) but not ≤ comparators (0%), while all other included tests had the opposite. The greatest rate was in *Nucleated red blood cell count* with 86% of results returned with a ≤ comparator, followed by *Quantitative faecal immunochemical test* (72%) and *Rheumatoid factor* (71%). The remaining tests had 29–40% of test results with comparators ( Table 2).

**Table 2.** Count and rate of comparators per test over the two-week study period. Counts are rounded to the nearest 10.

			Any comparator		<=	>=
Test type	SNOMED term	Total tests	n	%	%	%
GFR	Glomerular filtration rate	10,730	4,810	45%	0%	45%
	GFR calculated by abbreviated MDRD	138,710	45,560	33%	0%	33%
	EGFR calculated using creatinine CKDEC equation	203,290	63,690	31%	0%	31%
Other blood tests	Nucleated red blood cell count	21,690	18,580	86%	86%	0%
	Rheumatoid factor	6,120	4,340	71%	71%	0%
	Tissue transglutaminase immunoglobulin A level	9,060	3,590	40%	39%	0%
	Plasma C reactive protein	13,370	5,010	37%	37%	0%
Urine	Urine albumin level	30,880	9,640	31%	31%	0%
	Urine microalbumin level	14,440	4,180	29%	29%	0%
Faecal	Quantitative faecal immunochemical test	7,350	5,460	74%	72%	2%
	Faecal calprotectin content	5,790	1,940	34%	32%	1%

*(E)GFR = (Estimated) Glomerular filtration rate* *CKDEC = Chronic Kidney Disease Epidemiology Collaboration equation per 1.73 square metres* *MDRD = Modification of Diet in Renal Disease Study Group calculation*

### Values associated with comparators

For the included GFR tests, the most common results involving comparators were “≥60” and “≥90”, where 90–96% of tests with numeric value 60 or 90 had a ≥ comparator ( Table 3).

**Table 3.** Most common comparator results for each included GFR test. For each test, rows are divided into each of the commonly occurring “comparator” results e.g. “≥60”, “≥90”. Columns show the number of test results which appeared with the comparator, the total tests with the same numeric value with or without comparators (e.g. “60”, “90”), and the calculated percentage of results with comparators. Counts are rounded to the nearest 10. The most commonly appearing “lower reference limits” are also shown; results above these values are considered “normal”. These values are sometimes supplied alongside a test result to provide the appropriate reference limit for the patient (e.g. for their age group). The most common non-zero value is shown, alongside the percentage of tests to which they were attached.

	Results with comparator		Total results with corresponding value (with or without comparator)		% with comparator	Most common lower reference limit	
term	Result	n	Result value	n		value	% of total results
Glomerular filtration rate	≥60	3,050	60	3,190	96%	60.0	34%
	≥90	1,750	90	1,890	93%		
GFR calculated by abbreviated MDRD	≥90	32,210	90	33,830	95%	60.0	9%
	≥60	13,240	60	14,790	90%		
	≥120	110	120	130	85%		
EGFR calculated using creatinine CKDEC equation	≥90	63,690	90	66,230	96%	60.0	24%

*(E)GFR = (Estimated) Glomerular filtration rate* *CKDEC = Chronic Kidney Disease Epidemiology Collaboration equation per 1.73 square metres* *MDRD = Modification of Diet in Renal Disease Study Group calculation*

Values associated with comparators for the non-GFR tests are shown in Table 4. Nucleated RBC test results with values of 0.5 or 0.2 were almost always returned with ≤ comparators (99-100%). These corresponded to the common upper limit values (0.2 in 55% of tests and 0.5 in 31%).

**Table 4.** Most common comparator results for each included non-GFR test. For each test, rows are divided into each of the commonly occurring “comparator” results e.g. “≤0.2”, “≤0.5”. Columns show the number of test results which appeared with the comparator, the total tests with the same numeric value with or without comparators (e.g. “0.2”, “0.5”), and the calculated percentage of results with comparators. Counts are rounded to the nearest 10. The most commonly appearing “upper reference limits” are also shown; results above these values are considered “normal”. These values are sometimes supplied alongside a test result to provide an appropriate reference limit for the patient (e.g. for their age group). The two most common non-zero values are shown, alongside the percentage of results to which they were attached. Counts are rounded to the nearest 10.

	Results with comparator		Total results with corresponding value (with or without comparator)		% with comparator	Most common upper reference limits (% of total results)			
	*Result*	*n*	Result value	n		*value*	*%*	*value*	*%*
*Nucleated red blood cell * *count*	*≤0.5*	*6,610*	*0.5*	*6,630*	*100%*	*0.2*	*55%*	*0.5*	*31%*
	*≤0.2*	*11,970*	*0.2*	*12,060*	*99%*				
*Rheumatoid factor*	*≤8.4*	*210*	*8.4*	*210*	*100%*	*14.0*	*46%*	*15.0*	*10%*
	*≤20.0*	*1,010*	*20.0*	*1,030*	*98%*				
	*≤7.0*	*350*	*7.0*	*380*	*92%*				
	*≤10.0*	*1,930*	*10.0*	*2,120*	*91%*				
	*≤13.0*	*140*	*13.0*	*190*	*74%*				
	*≤12.0*	*180*	*12.0*	*260*	*69%*				
	*≤9.0*	*160*	*9.0*	*260*	*62%*				
	*≤11.0*	*160*	*11.0*	*280*	*57%*				
	*≤10.5*	*30*	*10.5*	*280*	*11%*				
*Tissue transglutaminase * *immunoglobulin A level*	*≤5.0*	*220*	*5.0*	*230*	*96%*	*6.9*	*26%*	*7.0*	*12%*
	*≤1.9*	*260*	*1.9*	*300*	*87%*				
	*≤0.5*	*1,180*	*0.5*	*1,680*	*70%*				
	*≤0.1*	*270*	*0.1*	*390*	*69%*				
	*≤1.0*	*1,300*	*1.0*	*1,980*	*66%*				
	*≤0.2*	*300*	*0.2*	*780*	*38%*				
	*≤2.0*	*10*	*2.0*	*170*	*6%*				
*Plasma C reactive protein*	*≤0.5*	*370*	*0.5*	*400*	*93%*	*10.0*	*48%*	*5.0*	*46%*
	*≤5.0*	*3,170*	*5.0*	*3,580*	*89%*				
	*≤1.0*	*1,370*	*1.0*	*2,100*	*65%*				
	*≤4.0*	*90*	*4.0*	*510*	*18%*				
*Urine albumin level*	*≤3.0*	*3,860*	*3.0*	*4,200*	*92%*	*0.0*	*79%*	*20.0*	*11%*
	*≤5.0*	*2,180*	*5.0*	*2,570*	*85%*				
	*≤3.2*	*540*	*3.2*	*640*	*84%*				
	*≤6.6*	*370*	*6.6*	*450*	*82%*				
	*≤7.0*	*1,600*	*7.0*	*2,000*	*80%*				
	*≤4.0*	*440*	*4.0*	*700*	*63%*				
	*≤10.0*	*350*	*10.0*	*630*	*56%*				
	*≤6.0*	*250*	*6.0*	*580*	*43%*				
*Urine microalbumin level*	*≤3.0*	*1,760*	*3.0*	*2,000*	*88%*	*0.0*	*84%*	*20.0*	*15%*
	*≤2.0*	*410*	*2.0*	*520*	*79%*				
	*≤5.0*	*1,120*	*5.0*	*1,550*	*72%*				
	*≤7.0*	*570*	*7.0*	*790*	*72%*				
	*≤6.0*	*270*	*6.0*	*550*	*49%*				
*Quantitative faecal* *immunochemical test*	*≤7.0*	*1,920*	*7.0*	*1,990*	*96%*	*10.0*	*46%*	*0.0*	*45%*
	*≤2.0*	*1,420*	*2.0*	*1,480*	*96%*				
	*≤10.0*	*930*	*10.0*	*970*	*96%*				
	*≤4.0*	*990*	*4.0*	*1,100*	*90%*				
	*≤6.0*	*40*	*6.0*	*120*	*33%*				
*Faecal calprotectin * *content*	*≤20.0*	*400*	*20.0*	*430*	*93%*	*50.0*	*52%*	*0.0*	*22%*
	*≤30.0*	*520*	*30.0*	*560*	*93%*				
	*≤26.0*	*370*	*26.0*	*400*	*93%*				
	*≤4.0*	*100*	*4.0*	*110*	*91%*				
	*≤15.0*	*100*	*15.0*	*130*	*77%*				
	*≤5.0*	*70*	*5.0*	*100*	*70%*				
	*≤10.0*	*60*	*10.0*	*100*	*60%*				

For Rheumatoid factor, various test result values between 7–13 and 20 were commonly associated with ≤ comparators, (11–100%; Table 4). This test typically had no lower reference limit (1% of tests had a lower limit of 10.0); the most common upper limits were 14.0 (46%) and 15.0 (10%).

Plasma C reactive protein had various values associated with ≤ comparators, between 0.5-5.0 (18-93%; Table 4). This test typically had no lower reference limit (13% of tests had a lower limit of 0.1); common upper limits were 10.0 (48%) or 5.0 (46%).

Urine albumin had various values commonly associated with ≤ comparators, between 3.0-10.0 ( Table 4). This test typically had no lower reference limit (3% of tests had a lower limit of 3.0) or upper limit (79%); the most common upper limit present was 20.0 (11%). Urine microalbumin tests occurred less frequently but showed a broadly similar pattern, with values of 2.0-7.0 commonly associated with ≤ comparators.

Quantitative faecal immunochemical test had various values commonly associated with ≤ comparators, between 2.0-10.0 (33–96%; Table 4). This test most commonly had an upper reference limit of 10.0 (46%) or no upper limit (45%). Faecal calprotectin content was similar, with values 4.0-30.0 associated with ≤ comparators (60–93%) and common upper limits 50.0 (52%) or none (22%).

### Variation by region

**Figure 1.**
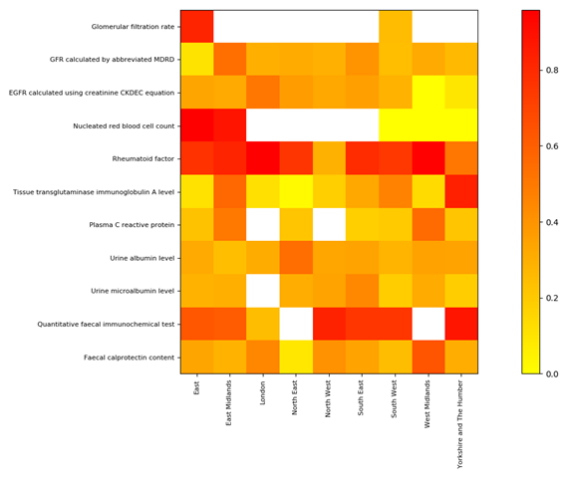
Comparator rates for each test by NHS region. Scale indicates the proportion of each test returned with a comparator (≥, >, ≤ or <) during the study period (1–14 October 2021. White squares represent denominators <100 tests. Only tests with non-zero result values are included. Values are shown in Table 5. Some regions do not have full population coverage in the cohort due to mixed practice software use: in particular London, the West Midlands and South East have less than 20% coverage in OpenSAFELY-TPP; North East and North West are less than 50% ^3^.

There was a large degree of variation on the use of comparators between regions for most tests ( Figure 1, Table 5), as well as the tests themselves, e.g. *Glomerular filtration rate* was only widely used in two of the nine regions ( Table 5). Each region had comparators present for at least some tests, and some tests showed particularly wide variation. For example, nucleated red blood cell counts had high rates of comparator usage in two regions (e.g. 88.3%, 11,970 of 13,560, East Midlands), but in the three other regions there were no comparators (however, denominators were much smaller (180-460).

**Table 5.** Comparator rates for each test by region. Results show the number of test results returned with a comparator, the total number of tests and the corresponding percentage. Counts are rounded to the nearest 10. Regions are excluded for individual tests where fewer than 100 total tests were recorded over the two week study period. Only tests with non-zero results are counted. Some regions do not have full population coverage in the cohort due to mixed practice software use: in particular London, the West Midlands and South East have less than 20% coverage in OpenSAFELY-TPP; North East and North West are less than 50% ^3^.

SNOMED code	SNOMED term	Region	Number of results with comparators	Total tests	%
80274001	Glomerular filtration rate	East	3,060	3,730	82%
		South West	1,750	6,910	25%
992081000000101	Rheumatoid factor	London	410	430	95%
		West Midlands	340	360	94%
		East Midlands	820	1,000	82%
		South East	190	240	79%
		East	1,180	1,540	77%
		North East	250	330	76%
		South West	460	620	74%
		Yorkshire and The Humber	510	990	52%
		North West	180	610	30%
999651000000107	Plasma C reactive protein	West Midlands	100	180	56%
		East Midlands	3,780	7,480	51%
		East	30	130	23%
		North East	340	1,550	22%
		Yorkshire and The Humber	30	140	21%
		South West	600	3,070	20%
		South East	130	720	18%
1002571000000102	Faecal calprotectin content	West Midlands	160	250	64%
		London	280	620	45%
		North West	160	390	41%
		South East	100	290	34%
		East	520	1,530	34%
		Yorkshire and The Humber	270	880	31%
		East Midlands	320	1,140	28%
		South West	120	480	25%
		North East	20	220	9%
1003301000000109	Urine albumin level	North East	250	460	54%
		West Midlands	300	860	35%
		Yorkshire and The Humber	2,110	6,130	34%
		South East	810	2,360	34%
		North West	820	2,460	33%
		East	2,150	6,750	32%
		London	930	2,970	31%
		South West	750	2,690	28%
		East Midlands	1,510	6,200	24%
1010251000000109	Urine microalbumin level	South East	260	580	45%
		North West	780	2,280	34%
		West Midlands	390	1,240	31%
		North East	660	2,170	30%
		East Midlands	740	2,450	30%
		East	800	2,800	29%
		South West	310	1,640	19%
		Yorkshire and The Humber	240	1,270	19%
1011481000000105	EGFR calculated using creatinine CKDEC equation	London	8,180	16,020	51%
		North East	4,850	13,080	37%
		South East	3,250	9,090	36%
		East	19,790	58,420	34%
		North West	2,700	8,210	33%
		East Midlands	14,320	44,600	32%
		South West	8,180	28,340	29%
		Yorkshire and The Humber	2,410	25,030	10%
		West Midlands	0	440	0%
1013671000000106	Tissue transglutaminase immunoglobulin A level	Yorkshire and The Humber	1,680	2,010	84%
		East Midlands	1,150	2,010	57%
		South West	140	300	47%
		South East	150	460	33%
		North West	100	550	18%
		West Midlands	70	510	14%
		London	20	170	12%
		East	280	2,580	11%
		North East	10	460	2%
1020291000000106	GFR calculated by abbreviated MDRD	East Midlands	17,080	31,560	54%
		South East	4,010	9,940	40%
		West Midlands	4,030	12,660	32%
		North East	1,420	4,490	32%
		London	260	860	30%
		North West	7,380	24,700	30%
		Yorkshire and The Humber	5,980	23,060	26%
		South West	3,570	14,540	25%
		East	1,770	16,770	11%
1022461000000100	Nucleated red blood cell count	East	6,610	6,900	96%
		East Midlands	11,970	13,560	88%
		Yorkshire and The Humber	0	380	0%
		South West	0	180	0%
		West Midlands	0	460	0%
1049361000000101	Quantitative faecal immunochemical test	Yorkshire and The Humber	920	1,050	88%
		North West	1,560	1,890	83%
		South East	430	570	75%
		South West	1,160	1,540	75%
		East	850	1,340	63%
		East Midlands	500	810	62%
		London	30	120	25%

## Discussion

### Summary

We identified 11 common test codes in primary care data where at least one in four results were returned as a range of values, as indicated by a comparator such as ≥ or ≤, which may impact how these test results are interpreted in health data research. Three of the affected tests were related to GFR testing (reduced GFR indicates reduced kidney function), with results commonly returned as “≥60” or “≥90” rather than exact values. The other eight tests (four blood tests, two urine and two faecal tests) were returned with ≤ comparators. Reassuringly, the most common values returned with comparators were typically lower than the associated lower reference limit so unlikely to have an impact on interpretation of these result values. Regional variation in the proportion of tests returned with comparators indicates different conventions between different testing laboratories for some tests.

### Strengths and weaknesses

Here we aimed to inform research using electronic health records to categorise patients’ health status on a population level; our results are not intended to be applicable to the clinical interpretation of individual patient’s test results, where the clinician would have richer contextual information at hand. We expect our results to be generalisable to primary care data held in other UK research platforms, as the results are returned from laboratories with comparators, where applicable. However, we only include practices using TPP software, which are geographically clustered, so it is possible that testing laboratories not covered by this analysis may have different conventions. There may be other factors which influence the use of comparators e.g. patient age or comorbidities, where these affect the clinical threshold between normal and abnormal results. We only included a small subset of tests, but they were the most common tests in our sampling period with at least 25% of results returned with comparators. We also only covered a short (two weeks) sample of test results. It is possible there could be seasonal effects on testing patterns or results at different times of the year ^4, 5^. Some additional information relating to test results may be supplied in free text which is not currently available in OpenSAFELY.

### Findings in context

Test results in electronic health records should always be interpreted with caution as “abnormal” results alone do not necessarily indicate pathology, and other information e.g. symptoms may be important to determine the patient’s condition, which may not be available to the researcher. This report adds further clarity on applying threshold values to classify patients’ health status based upon test results in electronic health records research.

For the GFR-related tests, we found results commonly returned as “≥60” or “≥90”. While 90+ typically represents healthy kidney function, values 60-<90 may represent mild kidney disease in combination with other signs or symptoms ^6^; therefore, patients with result “≥60” cannot be fully distinguished between these states. However, most commonly in health data research, and in OpenSAFELY research to date, categories are only created for moderate to severe disease (GFR <60), and these are unaffected by comparators. GFR values are also often recalculated from the raw creatinine values in electronic health records. However, some studies in the literature do make distinctions between the 60-<90 and 90+ groups ^7^.

For the other eight tests we identified, no OpenSAFELY studies to date have used result values from these tests without also returning the operator, but there are some implications for interpretation of these result values. Taking quantitative faecal immunochemical tests as an example, values ≥10 are typically classified as *abnormal* results, in combination with other information ^8^; however, we found the value 10 appeared with a “<” or “≤” symbol 96% of the time, indicating a *normal* result. Therefore, contrary to convention, 10 should be included as a normal result for this test, when analysing results in health record data.

### Implications

It is important to recognise when using laboratory test result values in electronic health records that a numeric value does not always represent an exact measurement result. One implication of the presence of comparators is that the absolute change in values over time within an individual cannot always be determined. In addition, there can be clusters of results at the same or similar values. Users of primary care data should check the comparators returned with test result values to ensure they are interpreting them correctly, especially for the tests identified in this report and for less common tests which we have not included here.

### Summary

Some test results values in electronic health records are commonly returned with comparators, indicating a range of possible values. For the common tests we identified, we expect there to be minimal implications for researchers for most use-cases. However, we found that for several GFR test codes, the result values cannot be accurately used to distinguish mild kidney disease from healthy kidney function. In general, users of test result values in electronic health records should take care when using threshold values to classify a patient’s condition and consider extracting any associated comparators alongside numeric values.

## Information governance and ethical approval

NHS England is the data controller of the NHS England OpenSAFELY COVID-19 Service; TPP is the data processor; all study authors using OpenSAFELY have the approval of NHS England ^9^. This implementation of OpenSAFELY is hosted within the TPP environment which is accredited to the ISO 27001 information security standard and is NHS IG Toolkit compliant ^10^.

Patient data has been pseudonymised for analysis and linkage using industry standard cryptographic hashing techniques; all pseudonymised datasets transmitted for linkage onto OpenSAFELY are encrypted; access to the NHS England OpenSAFELY COVID-19 service is via a virtual private network (VPN) connection; the researchers hold contracts with NHS England and only access the platform to initiate database queries and statistical models; all database activity is logged; only aggregate statistical outputs leave the platform environment following best practice for anonymisation of results such as statistical disclosure control for low cell counts ^11^.

The service adheres to the obligations of the UK General Data Protection Regulation (UK GDPR) and the Data Protection Act 2018. The service previously operated under notices initially issued in February 2020 by the Secretary of State under Regulation 3(4) of the Health Service (Control of Patient Information) Regulations 2002 (COPI Regulations), which required organisations to process confidential patient information for COVID-19 purposes; this set aside the requirement for patient consent ^12^. As of 1 July 2023, the Secretary of State has requested that NHS England continue to operate the Service under the COVID-19 Directions 2020 ^13^. In some cases of data sharing, the common law duty of confidence is met using, for example, patient consent or support from the Health Research Authority Confidentiality Advisory Group ^14^.

Taken together, these provide the legal bases to link patient datasets using the service. GP practices, which provide access to the primary care data, are required to share relevant health information to support the public health response to the pandemic, and have been informed of how the service operates.

This study was approved by the Health Research Authority (REC reference 20/LO/0651) and by the LSHTM Ethics Board (reference 21863).

## Data Availability

Access to the underlying identifiable and potentially re-identifiable pseudonymised electronic health record data is tightly governed by various legislative and regulatory frameworks, and restricted by best practice. The data in the NHS England OpenSAFELY COVID-19 service is drawn from General Practice data across England where TPP is the data processor. TPP developers initiate an automated process to create pseudonymised records in the core OpenSAFELY database, which are copies of key structured data tables in the identifiable records. These pseudonymised records are linked onto key external data resources that have also been pseudonymised via SHA-512 one-way hashing of NHS numbers using a shared salt. University of Oxford, Bennett Institute for Applied Data Science developers and PIs, who hold contracts with NHS England, have access to the OpenSAFELY pseudonymised data tables to develop the OpenSAFELY tools. These tools in turn enable researchers with OpenSAFELY data access agreements to write and execute code for data management and data analysis without direct access to the underlying raw pseudonymised patient data, and to review the outputs of this code. All code for the full data management pipeline — from raw data to completed results for this analysis — and for the OpenSAFELY platform as a whole is available for review at github.com/OpenSAFELY. The data management and analysis code for this paper was led by HJC. Contact helen.curtis@phc.ox.ac.uk.

## Software availability

- Code available from https://github.com/opensafely/pathology-comparators-short-report.
- Archived code at time of publication. https://doi.org/10.5281/zenodo.8325326
