# Supplementary material for: Interpreting pathology test result values with comparators (< , >) in Electronic Health Records research: an OpenSAFELY short data report
Source: Wellcome Open Res. 2024 Jul 2;8:541. Originally published 2023 Nov 22. [Version 2] doi: 10.12688/wellcomeopenres.19858.2 (PMC10904973; doi:10.12688/wellcomeopenres.19858.2)
Supplement: Peer review history

# Reviewer report: Reviewer response for version 2

**DOI:** 10.21956/wellcomeopenres.25001.r106886

**Published:** 2024-11-20

**Authors:** Hanieh Razzaghi

**Affiliations:** 1 The Children's Hospital of Philadelphia, Philadelphia, Pennsylvania, USA

In this work, the authors express the importance of considering comparators associated with numeric values when performing research on quantitative lab results. The authors included patients of all ages registered with a GP in OpenSAFELY-TPP and labs under study include GFR, nucleated red blood cell count, rheumatoid factor, tissue transglutaminase immunoglobulin A level, and plasma c reactive protein. The study is descriptive of current state and best practices but does not focus on the impact of neglecting comparators on currently published research and the importance of considering them for future research. While the paper identifies data quality issues for lab representation in EHRs, there is limited discussion on implications, provenance, and solutions. However, the paper describes challenges of working with secondary use of EHR data for research, and is a critical first step in furthering our understanding of laboratory data.

 Major issues:

- Introduction: The authors do not contextualize the study and should cite more literature. Is this research novel, or has it been described elsewhere? Is it unique to this database or do other EHR-sourced databases also have this issue? If this is completely novel research, the authors should still describe the current state of EHR as a source of secondary use in research and the range of studies dependent on accurate lab data representation.
- Codelist construction: it is not clear how the sample period was determined to identify the set of common tests with comparators. Was regional or seasonal variability considered in the sampling strategy, or were the tests selected skewed by healthcare systems with more robust testing data or specialties with higher utilization?
- Methods: Can the authors justify why they limited their analyses to a small number of labs? While examining the clinical implications of lab representation requires focusing on a smaller number of labs, the descriptive statistics can be scaled to include a much larger set of lab results.
- Methods: The authors should describe their data source in more depth. I am curious whether the underlying data provenance can be adjusted or fixed, or whether that is infeasible for the researchers. Are the results reported as a range from the lab, or is there a conversion elsewhere in the process? For example, is GFR reported this way in the data because the original serum creatinine value is also reported?
- Results: The results are lacking a clear description of the impact of this work in a scientific context. Without providing references to studies for which interpretation of the results could have been impacted by consideration of comparators, the reader must make assumptions about the potential negative impact of ignoring comparators.
- Results: Though the authors show the widespread use of comparators in a subset of lab tests with quantitative results, the results lack quantification of labs that would be misinterpreted in a way that would lead to misclassification of severe disease.

Minor issues:

- GP should be defined.
- I think the first sentence of the third paragraph of the introduction should read “we therefore set out to find the most affected tests, how often they have comparators, and *in* which direction.”
- It would be helpful to see, in a more straightforward way, the quantity of results for which interpretation in relation to limits of normal would change when taking into consideration the comparator. Table 3 hints at this, but the main takeaway should be “if the comparator was considered, x% of results would be considered abnormal that otherwise would be considered normal.
- In the Discussion, the authors discuss GFR values, and the importance of distinguishing between the 60-90 and 90+ range. It may be of value to compare these numbers to the original serum creatinine values, as eGFR is derived from serum creatinine and can recompute the original value if needed.
- In the Discussion, authors should expand on the clinical interpretation of these comparators. There is some discussion of the implications of 60-90 and 90+ GFR, but other labs are not discussed --- for example, is there a clinical difference between 0.5 and 0.2 in nucleated RBC?

Is the work clearly and accurately presented and does it cite the current literature?

Partly

If applicable, is the statistical analysis and its interpretation appropriate?

Yes

Are all the source data underlying the results available to ensure full reproducibility?

No source data required

Is the study design appropriate and is the work technically sound?

Partly

Are the conclusions drawn adequately supported by the results?

Yes

Are sufficient details of methods and analysis provided to allow replication by others?

Yes

Reviewer Expertise:

Informatics, Data Quality, Secondary use of EHR Data

I confirm that I have read this submission and believe that I have an appropriate level of expertise to confirm that it is of an acceptable scientific standard, however I have significant reservations, as outlined above.

# Reviewer report: Reviewer response for version 2

**DOI:** 10.21956/wellcomeopenres.25001.r104602

**Published:** 2024-10-29

**Authors:** Mariska M.G. Leeflang

**Affiliations:** 1 Department of Epidemiology and Data Science, Amsterdam UMC, University of Amsterdam, Amsterdam, The Netherlands

Thank you for the opportunity to review this manuscript. This is a sound study, transparently reported and with all the codes available. Still, as a relative outsider it is difficult to understand all the codes and even the output files. Having said that, most suggestions I have are really minor. The only more major point may be that the study is really descriptive and that it is not clear to what extent the described data are representative for the whole dataset and what the extent of the findings is.

 1. As a reader, the title and abstract are not immediately clear. Some terms may need rephrasing or explanation, in particular: 'comparators' (why not use 'comparator sign'?); and 'an OpenSAFELY short data report'. Adding a few words in the abstract to explain OPenSAFELY would also help.

  2. I am not sure of these are 'pathology test results'. These are just regular blood test, and later in the report the authors also call them 'laboratory test results'. To me, pathology tests refers more to cytology or histology test results. 

 3. It would be insightful if the authors could expand a bit more on the consequences of the methodological decisions they made: e.g. what may have been the consequences of ignoring free text information; and what may have been the consequences of only limiting the data to those practices with TPP? In the weaknesses section these issues are mentioned, but not their potential consequences.

 4. PPI is explained for the OpenSAFELY dataset as a whole, but not for this particular study. Please do.

 5. It would be insightful if the authors could expand a bit more on the findings. For example, how many tests are there in total and what proportion suffers from < and > use? How often is it problematic?

 6. Authors limit their findings to tests where 25% or more has a comparator. Could this have led to selection bias? 

 7. Results - Under "Overall rate of comparators present per test":  "(36.1%) of which had results that were associated with a comparator". I think a word is missing here,

Is the work clearly and accurately presented and does it cite the current literature?

Yes

If applicable, is the statistical analysis and its interpretation appropriate?

Yes

Are all the source data underlying the results available to ensure full reproducibility?

Partly

Is the study design appropriate and is the work technically sound?

Yes

Are the conclusions drawn adequately supported by the results?

Yes

Are sufficient details of methods and analysis provided to allow replication by others?

Yes

Reviewer Expertise:

Evaluations of medical tests; mainly diagnostic test accuracy

I confirm that I have read this submission and believe that I have an appropriate level of expertise to confirm that it is of an acceptable scientific standard.

# Reviewer report: Reviewer response for version 1

**DOI:** 10.21956/wellcomeopenres.21991.r71235

**Published:** 2024-02-26

**Authors:** Jessica Watson

**Affiliations:** 1 University of Bristol, Bristol, England, UK

This is a useful report for future users of OpenSAFELY which also has relevance for researchers using other Electronic Health Record databases to look at numeric test result values.

 Clear and transparent methods, including links to github code, are provided. The authors acknowledge the main limitation, which is that they only looked at a small subset of tests (with at least 25% of results returned with comparators). 

 In the discussion section 'findings in context' the authors describe the potential implications of these findings for researchers seeking to dichotomise continuous test results from OpenSAFELY into binary (normal/abnormal) results. This is an important issue, particularly for FIT tests as they have highlighted.

 However other issues such as the variation between different laboratories in cutoffs for 'abnormal' results, and issues with different units of measurement (eg for HbA1c and Hb) are probably a more prevalent problem for researchers when trying to categorise test results in this way. 

 I would also like the authors to consider the implications for researchers seeking to use continuous test results in analyses - for example summary measures of test results such as means, will be misleading if test results which include a comparator are treated as numeric results. This can usually be identified by exploring continuous results using a histogram to identify any unexpected peaks which might occur in association with a comparator.

 I would see this contextualised as part of a wider issue in EHR research, the importance of carefully exploring and understanding your data prior to analysis. I thoroughly support the OpenSAFELY team approach of standardising code to identify recurring issues such as this, and ensure transparency and rigor in EHR research.

Is the work clearly and accurately presented and does it cite the current literature?

Yes

If applicable, is the statistical analysis and its interpretation appropriate?

Yes

Are all the source data underlying the results available to ensure full reproducibility?

Yes

Is the study design appropriate and is the work technically sound?

Yes

Are the conclusions drawn adequately supported by the results?

Yes

Are sufficient details of methods and analysis provided to allow replication by others?

Yes

Reviewer Expertise:

General practitioner with expertise in diagnostic testing and electronic health records research.

I confirm that I have read this submission and believe that I have an appropriate level of expertise to confirm that it is of an acceptable scientific standard.

# Reviewer report: Reviewer response for version 1

**DOI:** 10.21956/wellcomeopenres.21991.r70395

**Published:** 2024-01-31

**Authors:** Tom Oates

**Affiliations:** 1 Barts Health NHS Trust, London, UK

Summary

 In this study the authors use the OpenSAFELY platform to retrospectively examine the EHR of 24m patients to find results returned with a comparator. 11 test codes showed >=25% of records with comparators. These tests encompassed blood, urine and faecal tests. GFR tests were returned with >= comparators and the remaining tests with =< comparators. Numeric data around how often these test results are returned with comparators and geographic variation in results is reported. 

 Major Issues

 - In the conclusions section of the abstract referring to GFR 60-90 as "mild kidney disease" is too vague; the word "mild" also appears in association with a GFR of 45-59 (see https://ukkidney.org/health-professionals/information-resources/uk-eckd-guide/ckd-stages). Perhaps change to "stage G2 CKD" 

 Minor Issues

 - might it be worth making the point specifically about the need to check for the presence of comparator values if using the tests identified as continuous variables in eg. prediction models?

 Typos/formatting

 - The final sentence of Methods>Data Source is duplicated

Is the work clearly and accurately presented and does it cite the current literature?

Yes

If applicable, is the statistical analysis and its interpretation appropriate?

Yes

Are all the source data underlying the results available to ensure full reproducibility?

Yes

Is the study design appropriate and is the work technically sound?

Yes

Are the conclusions drawn adequately supported by the results?

Yes

Are sufficient details of methods and analysis provided to allow replication by others?

Yes

Reviewer Expertise:

I'm an NHS consultant nephrologist with a PhD in genomics and some experience in large clinical data projects

I confirm that I have read this submission and believe that I have an appropriate level of expertise to confirm that it is of an acceptable scientific standard.
